# Effect of 2‐h hypoxic exposure on resting hepcidin levels in young adults

**DOI:** 10.14814/phy2.70530

**Published:** 2025-10-13

**Authors:** Chao‐an Lin, Masatoshi Naruse, Chihiro Tomiishi, Hikaru Matsudo, Claire E. Badenhorst, Kazushige Goto

**Affiliations:** ^1^ Graduate School of Sport and Health Science Ritsumeikan University Kusatsu Shiga Japan; ^2^ Research Organization of Science and Technology Ritsumeikan University Kusatsu Shiga Japan; ^3^ Research Fellow of Japan Society for Promotion of Science Chiyoda‐ku Tokyo Japan; ^4^ School of Sport, Exercise and Nutrition Massey University Auckland New Zealand

**Keywords:** erythropoietin, erythroferrone, hepcidin, hypoxic exposure, iron regulation

## Abstract

We evaluated the effects of acute hypoxic exposure on serum hepcidin levels. In a crossover design, 11 healthy individuals (9 men and 2 women) completed a 2‐h period of seated rest in a hypoxic (HYP; fraction of inspired oxygen [FiO_2_]: 12.5%) or normoxic control (CON; FiO_2_: 20.9%) environment in the morning. Following the environmental exposure, participants rested in a CON environment for 6 h. Venous blood samples were collected at baseline, immediately post‐exposure, and at 2‐, 4‐, and 6‐h post‐exposure. Serum erythropoietin (EPO) levels increased significantly over time in both conditions (*p* < 0.05). At 2‐h post‐exposure, the EPO response was significantly greater in HYP than in CON (*p* < 0.05). Serum erythroferrone (ERFE) levels did not differ significantly between the HYP and CON trials at any time point. Serum hepcidin levels increased significantly at 4‐ and 6‐h post‐exposure in both trials compared to baseline (*p* < 0.05). However, no significant differences in serum hepcidin levels were observed between the HYP and CON trials at any time point. These findings suggest that a 2‐h hypoxic exposure enhances the erythropoietic response in young adults but does not suppress diurnal serum hepcidin or elevate ERFE levels.

## 1 INTRODUCTION

Hepcidin is the key hormone regulating systemic iron homeostasis. Elevated hepcidin levels inhibit dietary iron absorption and iron recycling from macrophages to protect the body from iron overload. However, excessive elevations in hepcidin may increase the risk of iron deficiency (Ganz, 2013; Nemeth et al., 2004). Recent studies indicate that strenuous exercise elevates circulating hepcidin levels, and as a result, it is considered to be a contributing factor to the reduced iron stores frequently observed in physically active individuals (Barney et al., 2022; Peeling et al., 2009; Sim et al., 2013). Considering the indispensable role of iron in oxygen transport, iron deficiency can precipitate a reduction in endurance capacity, fatigue, and compromised athletic performance (Beard, 2001; Davies et al., 1982; Dellavalle & Haas, 2012; Keller et al., 2024; Sim et al., 2019). Consequently, interventions designed to attenuate hepcidin levels may enhance iron availability and could mitigate the performance declines associated with iron deficiency.

Resting hepcidin levels are influenced by several physiological factors. High iron stores and systemic inflammation, particularly mediated by interleukin‐6, promote hepcidin secretion (Ganz et al., 2008; Nemeth et al., 2004), whereas increased erythropoietic activity, such as following blood loss or exposure to a hypoxic environment, suppresses hepcidin expression (Nicolas et al., 2002). Notably, and despite these regulatory factors, at rest, hepcidin exhibits a distinct diurnal rhythm, characterized by a twofold to sixfold increase from 6:00 am to 3:00 pm, followed by a gradual decline into the evening (Kemna et al., 2007; Troutt et al., 2012). In the field of sport science, substantial research has focused on strategies to attenuate the post‐exercise elevations in hepcidin to optimize iron availability, including nutritional interventions (e.g., carbohydrate supplementation) and anti‐inflammatory strategies (Badenhorst et al., 2016; Díaz et al., 2015; McKay et al., 2021). However, few studies have examined whether these interventions, or other physiological stimuli such as hypoxia, can influence resting hepcidin levels and its natural diurnal variation.

Subsequently, a possible strategy that could be used to reduce resting hepcidin levels is exposure to a hypoxic environment. Hypoxia triggers an erythropoietic response to compensate for reduced oxygen availability (Haase, 2013). This response includes the renal release of erythropoietin (EPO), which promotes the proliferation and differentiation of erythroblasts in bone marrow. Additionally, hypoxia stimulates the production of erythroferrone (ERFE), a hormone that subsequently has been shown to suppress hepatic hepcidin synthesis (Arezes et al., 2018; Robach et al., 2021). Furthermore, within athletic populations, previous research has demonstrated that prolonged exposure to normobaric or hypobaric hypoxia, lasting from days to weeks, can suppress hepcidin levels and enhance iron absorption (Goetze et al., 2013; Govus et al., 2017).

Although the erythropoietic adaptations to prolonged hypoxic exposure are well‐documented, there is increasing interest in the physiological effects of acute hypoxic exposure. Recent studies suggest that even brief hypoxic exposure can stimulate EPO production (Turner et al., 2017; Wojan et al., 2021). However, studies on ERFE and hepcidin responses to acute hypoxic exposure in humans remain limited and have primarily investigated the effect on post‐exercise hepcidin activity (Badenhorst et al., 2014). Therefore, we investigated the effect of acute hypoxic exposure on resting hepcidin levels throughout the day. We hypothesized that a 2‐h hypoxic exposure would increase EPO and ERFE levels, subsequently suppressing the typical diurnal rise in resting hepcidin during the 6‐h post‐exposure period.

## 2 MATERIALS AND METHODS

### 2.1 Participants

In total, 11 healthy, nonsmoking individuals (9 men and 2 women) were enrolled. The participant cohort had a mean age, height, weight, and body fat percentage of 24 ± 3 years, 172.3 ± 6.1 cm, 64.8 ± 6.5 kg, and 14.5 ± 4.8%, respectively. The female participants were naturally menstruating and underwent the exposure within the first 10 days of their menstrual cycle. Participants with a history of hypoxic exposure within the preceding 2 months were excluded. Written informed consent was obtained from all participants following a comprehensive explanation of the experimental procedures and potential risks. The Ethics Review Committee for Medical and Health Research Involving Human Subjects at Ritsumeikan University, Japan approved the study protocols (BKC‐LSMH‐2023‐096). The study was performed in accordance with the Declaration of Helsinki.

### 2.2 Experimental design

In a single‐blind, randomized, crossover design, participants completed 2 h of seated rest in a hypoxic (fraction of inspired oxygen [FiO_2_]: 12.5%; HYP) or normoxic control (CON; FiO_2_: 20.9%; NOR) environment. Each trial was conducted on separate days, with a minimum washout period of 7 days between sessions. Prior to each trial, participants were instructed to abstain from alcohol, caffeine, and strenuous exercise for at least 24 h. In addition, they were required to record their meals and to consume identical meals on the day preceding each trial.

**FIGURE 1:**
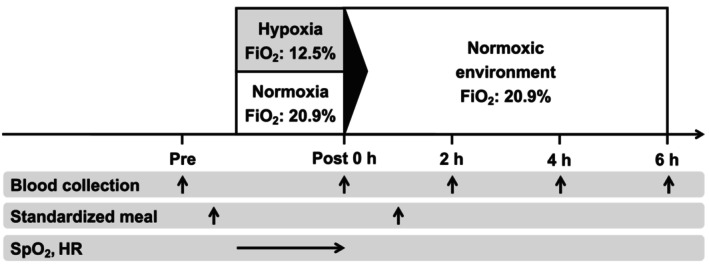
Experimental design. FiO_2_, fraction of inspiratory oxygen; HR, heart rate; SpO_2_, saturation of percutaneous oxygen. All hypoxic/normoxic control exposures started at 09:00 am.

All trials commenced at the same time of day to control for diurnal variations in EPO and hepcidin levels (Klausen et al., 1993, 1996; Troutt et al., 2012). Participants reported to the laboratory at approximately 08:00 am following a 10‐h overnight fast. Upon arrival, they rested for 5 min before baseline blood pressure was measured using an automated sphygmomanometer (HCR‐7201; Omron Healthcare Co., Ltd., Kyoto, Japan). Body composition was assessed using a multifrequency impedance technique (InBody 770; InBody Japan Inc., Tokyo, Japan). A venous blood sample was collected from the antecubital vein in a supine position between 08:20 and 08:30 am before breakfast. After the initial blood collection, participants consumed a standardized breakfast and then remained seated for 2 h inside an environmental chamber (Fuji Medical Science Co., Ltd., Chiba, Japan). All environmental exposures (normoxia or hypoxia) started between 9:00 and 9:10 am. During this period, participants were exposed to a hypoxic environment (HYP) or a normoxic environment (CON). Oxygen concentration (12.5% for HYP or 20.9% for CON), room temperature (23.0°C), and relative humidity (50%) were continuously monitored and automatically adjusted throughout the exposure period. Following the 2‐h exposure, participants transitioned to a normoxic environment for an additional 6 h of rest. Venous blood samples were collected immediately following the 2‐h exposure and at 2‐, 4‐, and 6‐h post‐exposure (Figure 1).

### 2.3 Experimental protocol

#### 2.3.1 Measurements of physiological variables and acute mountain sickness symptoms

During hypoxic or normoxic exposure, percutaneous oxygen saturation (SpO_2_) and heart rate (HR) were continuously monitored using a finger pulse oximeter (ATP‐W03; Fukuda Denshi Co., Ltd., Tokyo, Japan) placed on the tip of the forefinger. The recorded values were averaged every 30 min. Blood pressure was measured before entering the environmental chamber, at 30‐min intervals during the exposure period, and at 5 and 30 min after leaving the chamber. Participants were seated and allowed to rest for ≥5 min before each blood pressure measurement.

Acute mountain sickness (AMS) symptoms were assessed every 30 min during the hypoxic or normoxic exposure using the 2018 Lake Louise AMS Score (Roach et al., 2018). This scale evaluates headache, gastrointestinal discomfort, fatigue/weakness, and dizziness/light‐headedness, with each symptom rated on a 0–3‐point severity scale. All participants completed the hypoxic exposure. Mild headaches were reported by two participants 120 min after the onset of hypoxic exposure, but no other symptoms of AMS were observed.

#### 2.3.2 Dietary prescriptions

All participants recorded and consumed identical meals on the day before each trial. Meal records or photographs were submitted to the research team for verification. On the experimental day, standardized breakfast and lunch were provided as commercially prepackaged meals (Nissin Healthcare Food Service Co., Ltd., Tokyo, Japan). Participants were instructed to consume breakfast at 08:30 am and lunch at 12:15 pm. Water intake was permitted ad libitum. All meals were individually planned by a registered dietitian in accordance with the Dietary Reference Intakes for Japanese (2020) (Ministry of Health, Labour and Welfare of Japan, 2020), with the participants classified as having a low physical activity level. The macronutrient composition of the standardized meals (combined breakfast and lunch) was 14.1 ± 0.8% protein, 25.2 ± 1.4% fat, and 61.5 ± 0.8% carbohydrates. Energy and macronutrient intake per kilogram of body mass were 20.9 ± 0.8 kcal energy, 0.7 ± 0.0 g protein, 0.6 ± 0.0 g fat, and 3.2 ± 0.1 g carbohydrates.

#### 2.3.3 Blood sample and analyses

The first blood sample was collected using a butterfly needle and dispensed into a 2‐mL dipotassium ethylenediaminetetraacetic tube for whole blood and a 9‐mL serum separator tube for serum (Terumo Co., Tokyo, Japan). Subsequent blood samples, beginning with the collection immediately after exiting from the environmental chamber, were obtained via an indwelling venous cannula (Terumo Co., Tokyo, Japan) using a plastic syringe. Subsequently, these samples were dispensed into 6‐mL serum separator tubes.

Blood glucose levels were measured immediately after each blood collection using an automatic blood glucose analyzer (Freestyle; Nipro Co., Osaka, Japan), primarily to confirm the absence of hypoglycemia (no incidence was found). Serum separator tubes were allowed to clot for 30 min at room temperature before being centrifuged at 1710 *g* at 4°C for 10 min. Next, the serum supernatant was divided into 0.5‐mL aliquots and stored at −80°C until further analysis. Whole‐blood samples were refrigerated and subsequently sent to a clinical laboratory for further analysis.

Serum iron, ferritin, and EPO levels were measured from the obtained serum samples, whereas complete blood count parameters, including red blood cell count, hemoglobin, and hematocrit, were analyzed from whole blood samples at a clinical laboratory (SRL Inc., Tokyo, Japan). Serum hepcidin and ERFE levels were quantified using enzyme‐linked immunosorbent assay kits (hepcidin: Cat# DHP250, R&D Systems Inc., Minneapolis, MN, USA; ERFE: Cat# AG‐45B‐0014, Adipogen Life Sciences, San Diego, CA, USA). The intra‐assay coefficient of variation was 2.40% for hepcidin and 1.74% for ERFE.

### 2.4 Statistical analysis

All experimental data are presented as mean ± standard deviation. Time‐dependent changes in blood variables, SpO_2_, and HR were analyzed using a two‐way analysis of variance (ANOVA) with repeated measures, considering trial (HYP vs. CON) and time as factors. When a significant interaction or main effect was detected, a post hoc Tukey–Kramer test was conducted to identify specific differences. *p* values <0.05 were considered statistically significant.

## 3 RESULTS

### 3.1 Baseline hematological variables

Table 1 presents the baseline hematological variables. No statistically significant differences were observed between the HYP and CON trials for any of the measured hematological parameters (*p* > 0.05). Notably, one male participant exhibited a hemoglobin level < 13.5 g/dL, whereas two participants, one male and one female, presented with low iron storage (ferritin <15 ng/ mL). These individuals were retained in the analysis, as they still demonstrated measurable changes in EPO and hepcidin levels.

**TABLE 1:** Baseline hematological variables.

	HYP	CON
Red blood cell count (10^4^/μL)	481 ± 51	481 ± 42
Hemoglobin (g/dL)	14.4 ± 1.5	14.5 ± 1.2
Hematocrit (%)	43.8 ± 4.1	43.7 ± 3.3
Blood glucose (mg/dL)	92 ± 7	91 ± 7
Serum ferritin (ng/mL)	55.0 ± 32.9	55.8 ± 35.7
Serum iron (μg/dL)	107.8 ± 47.5	101.8 ± 44.1
Serum EPO (mIU/mL)	10.6 ± 3.4	9.9 ± 2.1
Serum ERFE (ng/mL)	4.65 ± 1.40	4.87 ± 1.28
Serum hepcidin (ng/mL)	20.70 ± 14.69	14.94 ± 12.93

*Note*: Values are means ± SD. No significant difference (*p* > 0.05) between trials was observed in all variables.

Abbreviations: CON, normoxic control trial; EPO, erythropoietin; ERFE, erythroferrone; HYP, hypoxia trial.

### 3.2 Physiological variables during HYP exposure

**FIGURE 2:**
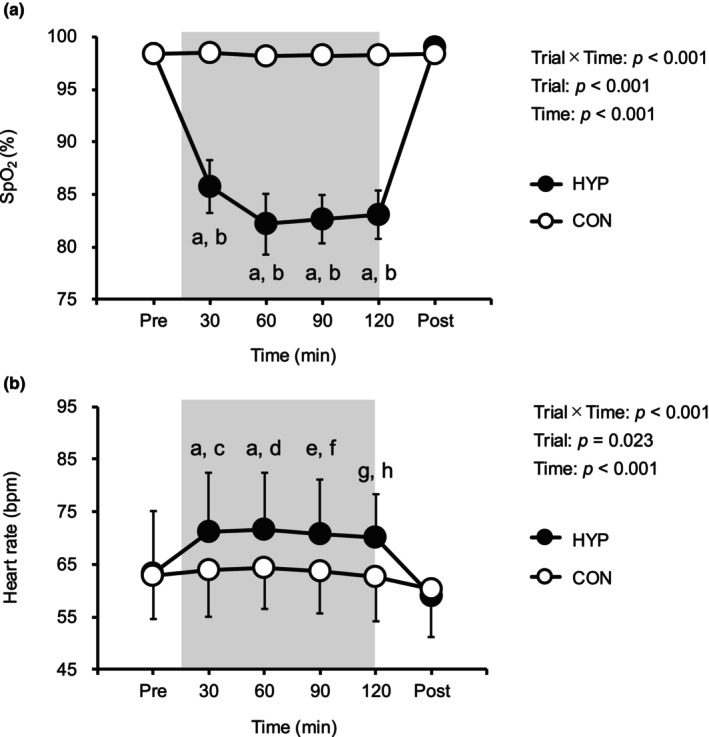
Changes in (a) SpO_2_ and (b) HR during 2 h of hypoxic or normoxic control exposure. The gray shaded area indicates hypoxic/normoxic control exposure period. Values are means ± SD. CON, normoxic control trial; HYP, hypoxia trial. CON, normoxic control trial. A: *p* < 0.001 versus Pre; B: *p* < 0.001 versus CON; C: *p* = 0.007 versus CON; D: *p* = 0.013 versus CON; E: *p* = 0.001 versus Pre; F: *p* = 0.01 versus CON; G: *p* = 0.002 versus Pre; H: *p* = 0.002 versus CON. Deviation bars for SpO_2_ in the normoxic control trial are not visible due to the small SD values (0.6%–0.8%).

SpO_2_ during hypoxic exposure was significantly lower in the HYP trial (83 ± 2%) compared to the CON trial (98 ± 1%, *p* < 0.05, Figure 2a). In addition, HR was significantly higher in the HYP trial (71 ± 12 bpm) compared to the CON trial (64 ± 9 bpm, *p* < 0.05, Figure 2b).

### 3.3 Serum EPO, ERFE, and hepcidin levels

**FIGURE 3:**
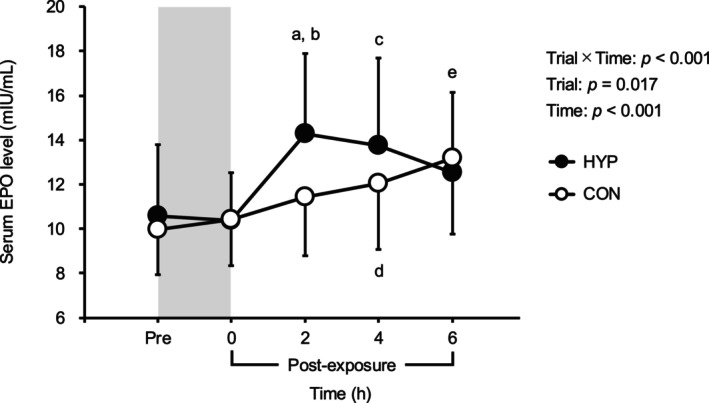
Changes in serum erythropoietin levels before and after hypoxic or normoxic control exposure. The gray shaded area indicates hypoxic/normoxic control exposure period. Values are means ± SD. CON, normoxic control trial; EPO, erythropoietin; HYP, hypoxia trial. A: *p* < 0.001 versus CON; B: *p* = 0.002 versus Pre; C: *p* = 0.009 versus Pre; D: *p* = 0.025 versus Pre; E: *p* = 0.004 versus Pre.

**FIGURE 4:**
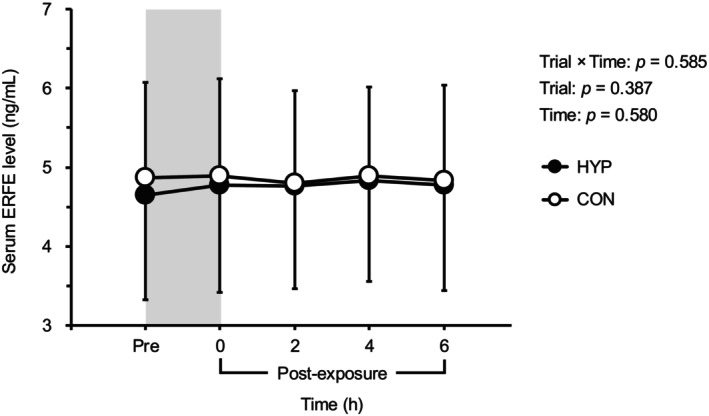
Changes in serum erythroferrone levels before and after hypoxic or normoxic control exposure. The gray shaded area indicates hypoxic/normoxic control exposure period. Values are means ± SD. CON, normoxic control trial; ERFE, erythroferrone; HYP, hypoxia trial.

Figure 3 illustrates the changes in serum EPO levels before and after hypoxic exposure. Serum EPO levels increased at 2, 4, and 6 h after hypoxic and normoxic exposure compared to baseline (*p* < 0.05). Notably, at 2 h post‐hypoxic exposure, serum EPO levels were significantly higher in the HYP trial (40.5 ± 31.9% increase from baseline) compared to the CON trial (16.1 ± 19.8% increase from baseline, *p* < 0.001). Serum ERFE levels did not differ between the two trials at any time point (*p* > 0.05, Figure 4).

**FIGURE 5:**
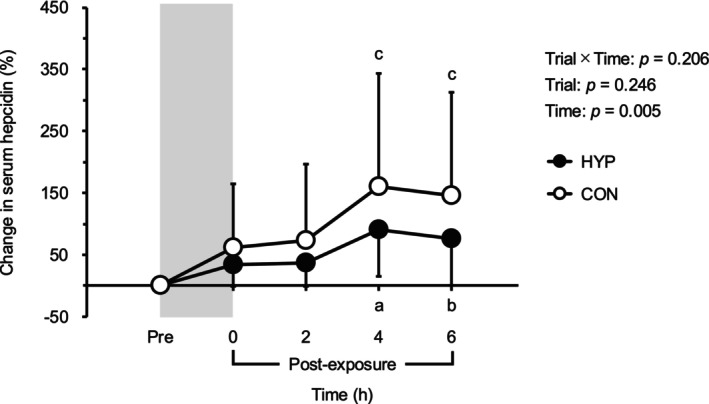
Percentage differences from baseline in serum hepcidin levels after hypoxic or normoxic control exposure. The gray shaded area indicates hypoxic/normoxic control exposure period. Values are means ± SD. CON, normoxic control trial; HYP, hypoxia trial. A: *p* = 0.004 versus Pre; B: *p* = 0.009 versus Pre; C: *p* = 0.02 versus Pre.

Figure 5 presents the relative changes in serum hepcidin levels. Hepcidin levels increased at 4 and 6 h after hypoxic or normoxic exposure compared to baseline (*p* < 0.05). However, no significant differences in serum hepcidin levels were observed between the two trials at any time point (*p* > 0.05).

## 4 DISCUSSION

To our knowledge, this is the first study to investigate the effect of acute hypoxic exposure on resting hepcidin levels in young, healthy individuals. The primary finding was that a 2‐h hypoxic exposure did not suppress the diurnal hepcidin response during the 6‐h post‐exposure period, despite successfully eliciting an erythropoietic response. Furthermore, the 2‐h hypoxic exposure did not increase serum ERFE levels.

The increase in EPO production under hypoxic conditions plays a crucial role in enhancing hematological variables (Berglund et al., 2002; Jelkmann, 2011). Previous studies have demonstrated that a 2‐h hypoxic exposure at an FiO_2_ of 10.5%–12.5% increases serum EPO levels by 43%–85% (Knaupp et al., 1992; Turner et al., 2017; Wojan et al., 2021). Consistent with these prior studies, we observed a 40.5 ± 31.9% increase in serum EPO levels at 2‐h post‐hypoxic exposure. Compared to the diurnal variations observed in the CON trial, this erythropoietic response in the HYP trial would appear to have been facilitated by the acute hypoxic exposure provided in our study. Despite the increase in serum EPO levels following the acute hypoxic exposure, our results demonstrated significant interindividual variability in EPO levels that is in alignment with what has been reported in previous studies (range: −0.9% to 89.2% from baseline) (Baranauskas et al., 2022; Chapman et al., 1998; Ge et al., 2002). This level of interindividual variability in EPO levels could possibly be attributed to individual differences in arterial oxygen saturation (SaO_2_), though research findings in this area are still inconclusive. Some researchers have reported a modest correlation between EPO and SaO_2_ (*r* = 0.41, *p* < 0.05), with no significant relationship observed between EPO and renal O_2_ delivery after 24 h of exposure to altitudes ranging from 1780 to 2800 m (Ge et al., 2002). While others have reported no significant correlation between SpO_2_ changes during hypoxic exposure and EPO levels across different simulated altitudes (3600 m, 4200 m, and 4800 m; *r* = − 0.11, *p* > 0.05) (Turner et al., 2017). Our study adds to this research and found no significant correlation between the mean SpO_2_ during 2‐h hypoxic exposure and subsequent changes in EPO levels (rs = −0.16, *p* > 0.05). In addition to oxygen delivery, the roles of genetic background (Jedlickova et al., 2003), systemic inflammation (Ganz, 2019; Weiss et al., 2019), and sex differences (Raberin et al., 2024) have also been discussed. However, we are not currently able to reach definitive conclusions. Based on the conflicting results, further research may be required to explore new related factors (e.g., other proinflammatory cytokines) that may contribute to the interindividual differences in EPO that are consistently reported with hypoxic exposure.

Several studies have demonstrated significantly suppressed resting hepcidin levels within 24–72 h following hypoxic exposure, with this suppression commonly preceded by EPO induction (Govus et al., 2017; Piperno et al., 2011; Ravasi et al., 2018). Initially, a direct inhibitory effect of EPO on hepcidin expression in hepatocytes was posited (Pinto et al., 2008). However, subsequent studies clarified that EPO's inhibitory effect on hepcidin was indirect and primarily mediated by its stimulation of ERFE production in erythroblasts (Arezes et al., 2018; Gammella et al., 2015; Kautz et al., 2014). Subsequently, we hypothesized that hypoxic exposure would attenuate the diurnal increase in serum hepcidin during the 6‐h post‐exposure period. Within the CON trial, serum hepcidin exhibited diurnal variations, increasing by 160 ± 183% from 08:00 am to 5:00 pm, results that would be consistent with previous studies (Kemna et al., 2007; Troutt et al., 2012). Yet, contrary to our initial hypothesis, the 2‐h hypoxic exposure did not suppress serum hepcidin levels, despite a substantial elevation in EPO levels (40.5 ± 31.9%). In addition, our results indicated no diurnal variations in serum ERFE levels between 08:00 am and 5:00 pm in either the CON or HYP trials and no change in serum ERFE levels following 2 h of hypoxic exposure. Given that no changes were found between trials for this key factor that is known to downregulate hepcidin activity, the absence of any hepcidin attenuation following hypoxic exposure and despite increases in EPO may be expected. Previous studies have reported that longer durations of hypoxic exposures, such as 15 h at high altitude (3800 m), may be required to increase ERFE levels (Robach et al., 2021). Similarly, sustained elevations in EPO through recombinant EPO injection have been shown to elevate serum ERFE and subsequently downregulate hepcidin 48–72‐h post‐administration. Therefore, while a 2‐h hypoxic exposure was sufficient to stimulate EPO secretion, this acute hypoxic exposure may have been insufficient to induce measurable changes in ERFE and hepcidin levels at rest.

Consistently throughout the literature, the iron status of the individual has been identified as the key determinant of hepcidin activity. Similarly, our study observed a strong positive correlation between baseline serum ferritin and hepcidin concentrations (rs = 0.83, *p* < 0.001). Results that would add to the current literature and reinforce the positive relationship between serum ferritin and hepcidin levels (Peeling et al., 2014; Walters et al., 1973). Within our study, two participants (one male and one female) with low serum ferritin concentrations (< 15 ng/mL) exhibited diminished baseline hepcidin levels (1.8 ng/mL and 2.9 ng/mL, respectively) and attenuated diurnal variations in hepcidin compared to participants with adequate ferritin levels (17.7 ± 11.9 ng/mL, *n* = 8). These findings further emphasize the importance and primary role of iron status in regulating hepcidin activity.

Interestingly, high‐altitude exposures of 3400–5400 m have typically produced a rapid decline in serum hepcidin and iron within 2–4 days (Goetze et al., 2013; Piperno et al., 2011; Talbot et al., 2012). However, in the present study, acute hypoxic exposure did not have a similar effect on iron status or hepcidin levels. No significant correlations were found between baseline serum ferritin levels and the percentage change in hepcidin levels at any time point within the study (*p* > 0.05). Additionally, the percentage change in hepcidin levels did not differ between trials (*p* > 0.05). Given the absence of hepcidin suppression, substantial mobilization of stored iron (ferritin) would not be expected, and a gradual increase in hepcidin throughout each trial could suggest potential iron sequestration at trial completion. On the other hand, adequate iron availability is known to support an optimal EPO response and facilitate erythropoietic adaptations to hypoxia at high altitudes (Gassmann & Muckenthaler, 2015; Okazaki et al., 2019). Interestingly, within our study, baseline serum iron concentrations were significantly correlated with the serum EPO concentration 2 h after hypoxic exposure (rs = 0.70, *p* = 0.02). Notably, although three participants exhibited low hemoglobin concentration or low iron stores, all of them maintained normal serum iron levels (> 50 μg/dL). These findings suggest that the variation in EPO levels was not strongly influenced by serum iron levels status. These findings could suggest that circulating serum iron may play a more significant role than stored iron in enhancing erythropoietic activity in response to acute hypoxic exposure. Such results would support current recommendations that aim to ensure adequate serum iron levels through dietary adjustments or supplementation for individuals undergoing high‐altitude training or artificial hypoxic exposure for positive hematological adaptations.

Our study has several limitations. First, the relatively small sample size limited the validity of subgroup analyses, making it challenging to identify factors associated with hepcidin secretion responsiveness following acute hypoxic exposure. Second, we investigated only the effects of a single bout of hypoxic exposure. Sustained intermittent hypoxic exposure has been shown to increase EPO synthesis, leading to a progressive rise in hemoglobin concentration (Rodríguez et al., 2000). However, the underlying mechanisms, as well as more efficient and safer protocols for long‐term intermittent hypoxic exposure, warrant further investigation. Finally, we assessed changes in the diurnal variation of hepcidin only at rest. Given the relevance to endurance athletes with iron deficiency, further studies are needed to investigate the effects of pre‐ or post‐exercise hypoxic exposure on hepcidin secretion in response to endurance exercise.

Despite these limitations, the present study offers a unique perspective. Most previous studies have focused on hypoxic exposure during exercise. In contrast, our findings suggest that an acute hypoxic stimulus at rest can stimulate an EPO response without adversely affecting iron regulation. Furthermore, we found that ERFE is a stable hormone that does not exhibit diurnal variation during the daytime and is not affected by a 2‐h hypoxic stimulus. To effectively investigate the “EPO–ERFE–hepcidin axis” using hypoxic exposure, a longer duration of hypoxia may be required to trigger ERFE secretion. This approach could align with “live high, train low” strategies, where athletes might perform acute hypoxic exposures on rest days to promote erythropoietic adaptations without compromising iron regulation.

## 5 CONCLUSION

A 2‐h hypoxic exposure stimulated EPO secretion in young men and women. However, despite this enhanced erythropoietic response, serum hepcidin levels and iron status did not decrease following hypoxic exposure. In addition, the 2‐h hypoxic exposure did not lead to an increase in serum ERFE levels. These results may suggest that acute hypoxic exposure at rest could be a beneficial strategy for inducing erythropoietic and hematological adaptations without adversely impacting an individual's iron status or increasing the risk of iron deficiency.

## AUTHOR CONTRIBUTIONS

CL and KG were part of the conception and protocol design. CL, MN, CT, and HM conducted the experiments. CL and KG were responsible for data analysis. CL and KG wrote the manuscript. CEB critically revised the manuscript and gave advice for corrections to CL.

## FUNDING INFORMATION

This study was supported in part by a grant from the Sasakawa Scientific Research Grant from The Japan Science Society to Chao‐an Lin.

## CONFLICT OF INTEREST STATEMENT

The authors declare no conflict of interest and have no financial relationship to disclose.

## ETHICS STATEMENT

The study protocols were approved by the Ethics Review Committee for Medical and Health Research Involving Human Subjects at Ritsumeikan University, Japan (Approval No. BKC‐LSMH‐2023‐096). All procedures were conducted in accordance with the Declaration of Helsinki.

## Data Availability

The data and analyses that support the findings of this study are not publicly available. The datasets are available from the corresponding author on reasonable request.
